# Age- and Sex-Dependency of Laser Speckle Flowgraphy Measurements of Optic Nerve Vessel Microcirculation

**DOI:** 10.1371/journal.pone.0148812

**Published:** 2016-02-12

**Authors:** Naoko Aizawa, Hiroshi Kunikata, Fumihiko Nitta, Yukihiro Shiga, Kazuko Omodaka, Satoru Tsuda, Toru Nakazawa

**Affiliations:** 1 Department of Ophthalmology, Tohoku University Graduate School of Medicine, Sendai, Japan; 2 Department of Retinal Disease Control, Tohoku University Graduate School of Medicine, Sendai, Japan; 3 Department of Advanced Ophthalmic Medicine, Tohoku University Graduate School of Medicine, Sendai, Japan; Harvard Medical School, UNITED STATES

## Abstract

**Purpose:**

To investigate the relationship between various characteristics of a normal population and laser speckle flowgraphy (LSFG) measurements of mean blur rate (MBR) in the optic nerve head (ONH).

**Methods:**

A total of 189 eyes of 189 normal subjects (93 male, 96 female, mean age 45 ± 14 years old, age range: 20–72) without any history of hypertension, hyperlipidemia or diabetes were enrolled. ONH microcirculation was measured with LSFG and overall MBR (MA), vessel-area MBR (MV), and tissue-area MBR (MT) were derived from these measurements. The statistical association of these measurements with characteristics such as sex, age, intraocular pressure (IOP) and systolic blood pressure (SBP) was then determined.

**Results:**

There was a trend towards decreased IOP and MV and increased SBP with age (*P* = 0.002, *P* = 0.035, and *P* = 0.006, respectively). Furthermore, IOP, MV and SBP were correlated with age (r = -0.23, *P* = 0.011; r = -0.24, *P* < 0.001; and r = 0.30, *P* < 0.001, respectively). Separate multiple regression analyses of independent contributing factors revealed that sex and IOP contributed to MA (*P* < 0.001 and *P* = 0.002, respectively), sex, IOP, and age contributed to MV (*P* < 0.001, *P* = 0.003, and *P* = 0.024, respectively), while only IOP contributed to MT (*P* = 0.003).

**Conclusion:**

In a normal population, MBR was affected by IOP in both the large vessel and capillary areas of the ONH, but not by SBP. MV was also affected by age and sex, while MT was stable independent of age or sex.

## Introduction

Ocular diseases related to ischemia, such as diabetic retinopathy, and ocular diseases related to circulatory disturbances and axonal damage, such as glaucoma, are the main causes of blindness worldwide.[1,2,3,4] They are particularly common in industrialized countries, and their successful treatment is a goal of public health. However, the pathogenesis of these blood flow-related ocular diseases remains unclear. It is therefore important to find new ways of studying these diseases and identifying them in patients. This calls for the development of new, non-invasive methods to measure ocular blood flow in living eyes.

Laser speckle flowgraphy (LSFG) is a promising candidate for such a method. It uses the laser speckle phenomenon to detect and analyze ocular blood flow, and can quantify ocular circulation *in vivo*. A recently developed LSFG measurement parameter, mean blur rate (MBR), can serve as a quantitative index of retinal blood cell speed. An earlier study showed that MBR was closely correlated to hydrogen gas clearance-based measurements of capillary blood flow (CBF) in the optic nerve head (ONH) of albino rabbits.[5] Another study found that MBR in the ONH of rhesus monkeys, with or without experimentally induced glaucoma, was closely correlated with microsphere-based measurements of CBF.[6] Clinically, MBR has been shown to be a highly reproducible, inter-individually comparable parameter, useful in evaluating the effect of medical interventions, grading the severity of glaucoma, and confirming the presence of ocular ischemic diseases.[7,8,9,10,11,12,13] However, the question still remains as to what effect individual characteristics such as age, sex, intraocular pressure (IOP) and blood pressure have on measurements of MBR. Additionally, the impact on MBR of age-related alterations in blood flow in the vascular and tissue regions of the ONH has not yet been determined.[14]

Vascular changes such as arterial stiffening are an inevitable part of aging that can affect many clinical biomarkers. In order to determine the precise effect of these changes on LSFG measurements of MBR, we were careful to select normal subjects who ranged widely in age. We divided these subjects into groups by decade of life, analyzed how MBR varied between each group, and determined the potential of MBR as a normative value for inter-individual and inter-group comparisons.

## Materials and Methods

### Setting and Patients

This retrospective study included 189 eyes of 189 healthy Japanese individuals (93 male, 96 female; mean age 45.4 ± 13.8 years old (YO); age range 20–74 YO) (Table 1) recruited from patients undergoing standard medical check-ups, i.e., internal medical check-ups, at Sendai Open Hospital. The exclusion criteria were: clinical evidence or history of ocular disease other than mild cataract or refractive error; evidence from optical coherence tomography or fundus photography suggesting glaucoma, i.e., abnormal loss of circumpapillary retinal nerve fiber layer thickness (cpRNFLT) or a cup-to-disc ratio of more than 0.4, respectively; history of intraocular surgery; presence of blood flow-affecting systemic disease requiring medical treatment; IOP more than 21 mmHg; and hypertension, hyperlipidemia, or diabetes. This study followed the tenets of the Declaration of Helsinki. All subjects gave written informed consent for their participation. The Ethics Committee of Sendai Open Hospital and the institutional review board of Tohoku University Graduate School of Medicine approved the research for this study.

**Table 1:** Characteristics of healthy subjects.

	All	Age					*P* value		
		20–29	30–39	40–49	50–59	≥ 60 years	50–59 vs. 20–29	≥ 60 years vs. 20–29	Trend for age
Number of eyes	189	36	29	34	63	27	-	-	-
Number of patients	189	36	29	34	63	27	-	-	-
Age (years)	45 ± 14	25 ± 3	35 ± 3	45 ± 3	54 ± 3	65 ± 5	-	-	-
Sex (M: F)	93: 96	16: 20	10: 19	18: 16	31: 32	18: 9	0.648^a^	0.115^a^	0.233^a^
IOP (mmHg)	13.8 ± 2.5	15.0 ± 2.1	14.3 ± 2.6	12.3 ± 2.0	14.0 ± 2.8	12.7 ± 2.1	0.511	0.007	0.002
SE (diopter)	-1.9 ± 2.1	-1.9 ± 1.6	-2.7 ± 1.8	-2.2 ± 2.0	-1.9 ± 2.3	-0.9 ± 2.0	1.000	0.246	0.082
SBP (mmHg)	118.8 ± 15.6	113.6 ± 13.5	113.8 ± 14.9	115.2 ± 15.0	120.4 ± 13.3	128.0 ± 19.4	0.254	0.046	0.006
DBP (mmHg)	71.7 ± 12.0	69.1 ± 10.8	71.5 ± 10.5	68.4 ± 12.3	73.5 ± 12.1	74.2 ± 13.3	0.596	0.727	0.283
MV (AU)	51.8 ± 6.8	54.1 ± 6.3	52.6 ± 8.4	52.0 ± 7.7	51.5 ± 6.2	48.7 ± 4.2	0.259	0.003	0.035
MT (AU)	13.5 ± 2.0	13.0 ± 2.1	13.2 ± 2.3	13.6 ± 1.8	13.8 ± 2.2	13.9 ± 1.5	0.430	0.277	0.297
MA (AU)	29.0 ± 4.2	30.5 ± 4.2	29.0 ± 4.9	28.7 ± 4.6	28.6 ± 3.9	27.9 ± 3.2	0.124	0.059	0.140

DBP = diastolic blood pressure, IOP = intraocular pressure, MA = overall mean blur rate, MT = mean blur rate in tissue area, MV = mean blur rate in vessel area, SBP = systolic blood pressure, SE = spherical equivalent

Unmarked P value: Steel-Dwass test, a: Chi-square test

### Physical and Ophthalmological Measurements

Systolic blood pressure and diastolic blood pressure (SBP and DBP) were measured after the patients had rested in a sitting position for 10 min. Measurements were made in the left brachial artery at the height of the heart by an automated blood pressure monitor (HEM-759E, Omron Corporation, Kyoto, Japan) before LSFG was used to measure ONH circulation. Ophthalmological examinations included fundus photography, measurement of IOP, spherical equivalent (SE), diopter (D), and optical coherence tomography (OCT) measurements of cpRNFLT (3D-OCT2000; TOPCON Corporation, Tokyo, Japan).

### Laser Speckle Flowgraphy Measurements

Before LSFG measurement, the pupils of each subject were dilated with 0.5% tropicamide and 0.5% phenylephrine hydrochloride. The details of the underlying principles of LSFG (Softcare, Fukutsu, Japan) have been described in previous reports.[15,16] Briefly, the LSFG device consists of a fundus camera equipped with a diode laser (wavelength 830 nm) and a camera. MBR (measured in arbitrary units; AU), the relative speed of blood flow, is derived from the pattern of speckle contrast produced by the interference of a laser scattered by blood cells moving in the ocular fundus.[17] MBR images are acquired continuously at the rate of 30 frames per second for a 4-second time period and transferred to a computer file. Integrated analysis software then synchronizes the captured MBR images in each cardiac cycle, and averages the MBR in each heartbeat to produce a heartbeat map of the ONH. The LSFG software automatically divides this map into the large vessel and capillary areas, and blood flow parameters are assessed separately for the vessel area (referred to as MV, “mean value vessel area”), the tissue area (referred to as MT, “mean value tissue area”) and the total area of the ONH (MA, “mean value all areas”). The statistical analysis was based on values for ONH circulation that represented the average of three measurements made with LSFG.

### Statistical Analysis

The Steel-Dwass test and chi-square test were used to determine the significance of differences between groups and trends related to age. Spearman’s rank correlation test was used to evaluate single correlations between age and each measurement variable (i.e., SE, IOP, SBP, DBP, MV, MT and MA). A multiple linear regression analysis was performed to determine independent variables affecting MT. The statistical analyses were performed with JMP software (Pro version 10.0.2, SAS Institute Japan Inc., Tokyo, Japan). The significance level was set at *P* < 0.05.

## Results

**Fig 1:**
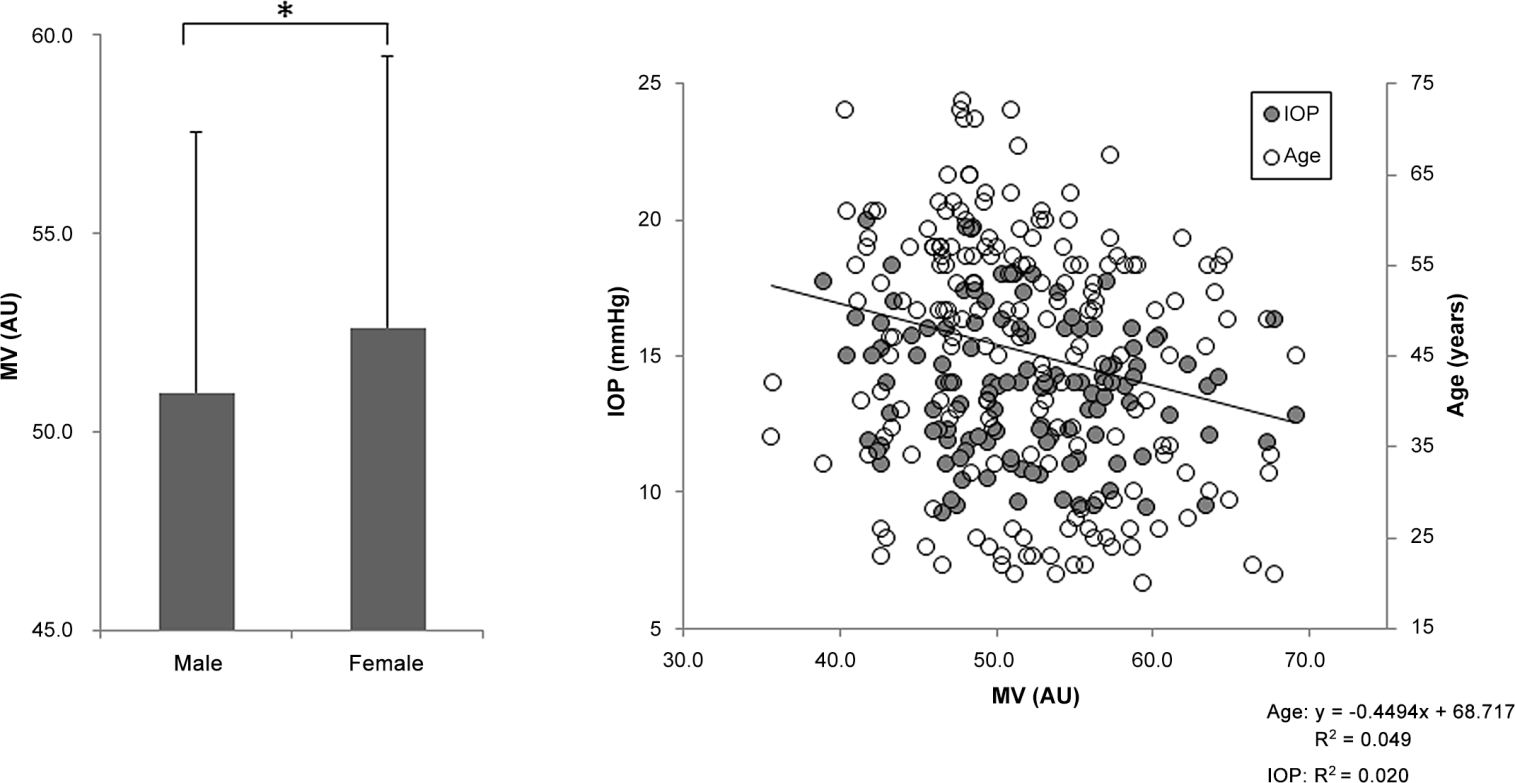
Relationship between vessel-area mean blur rate (MV) and clinical findings. MV was higher in the female subjects than in the male subjects (*P* < 0.05, left). MV was weakly correlated with age (r = -0.24, *P* < 0.001, right).

**Fig 2:**
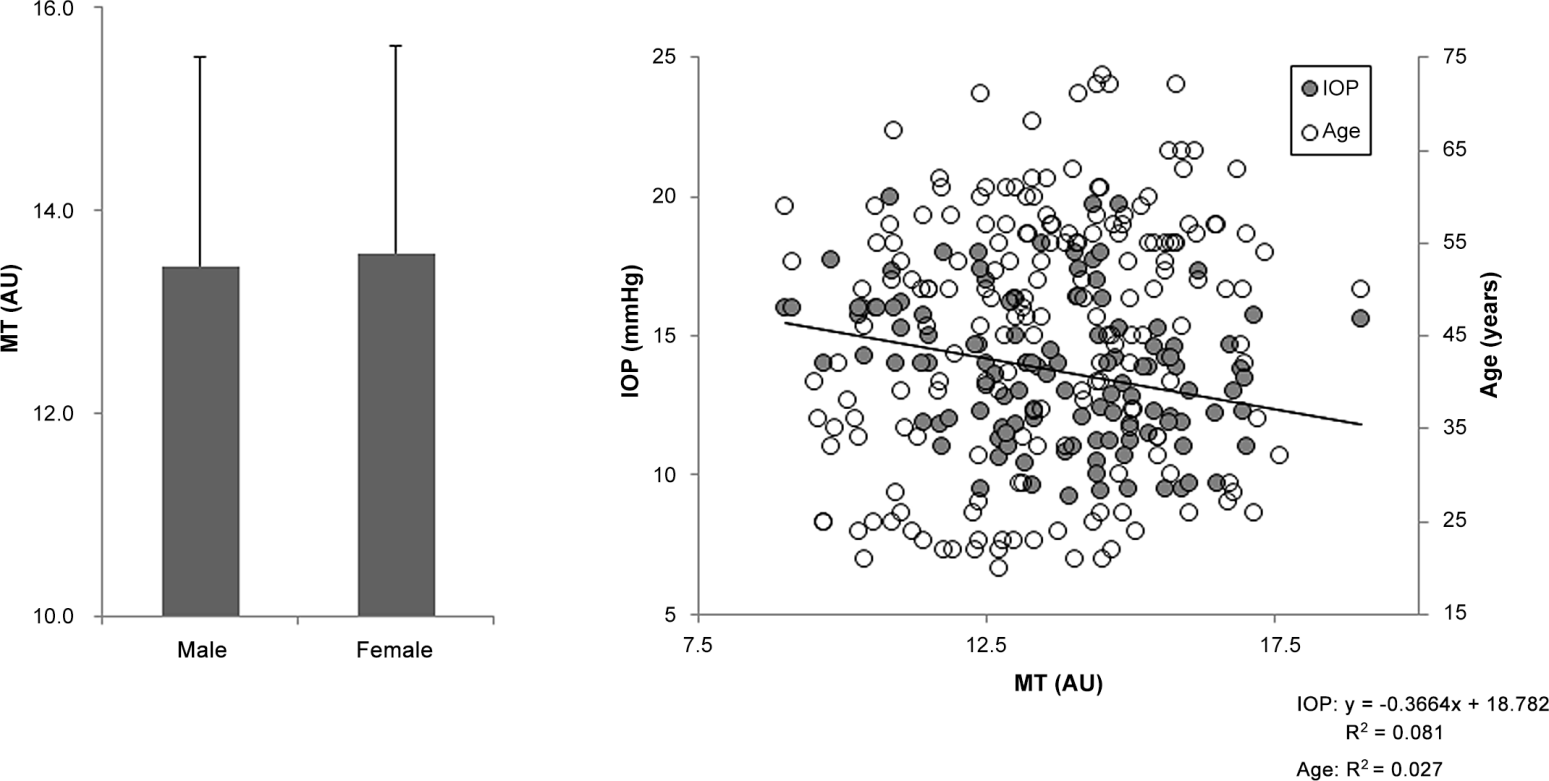
Relationship between tissue-area mean blur rate (MT) and clinical findings. There was no significant difference in MT between the female and male subjects (left). MT was weakly correlated with IOP (r = -0.29, *P* = 0.001, right).

**Fig 3:**
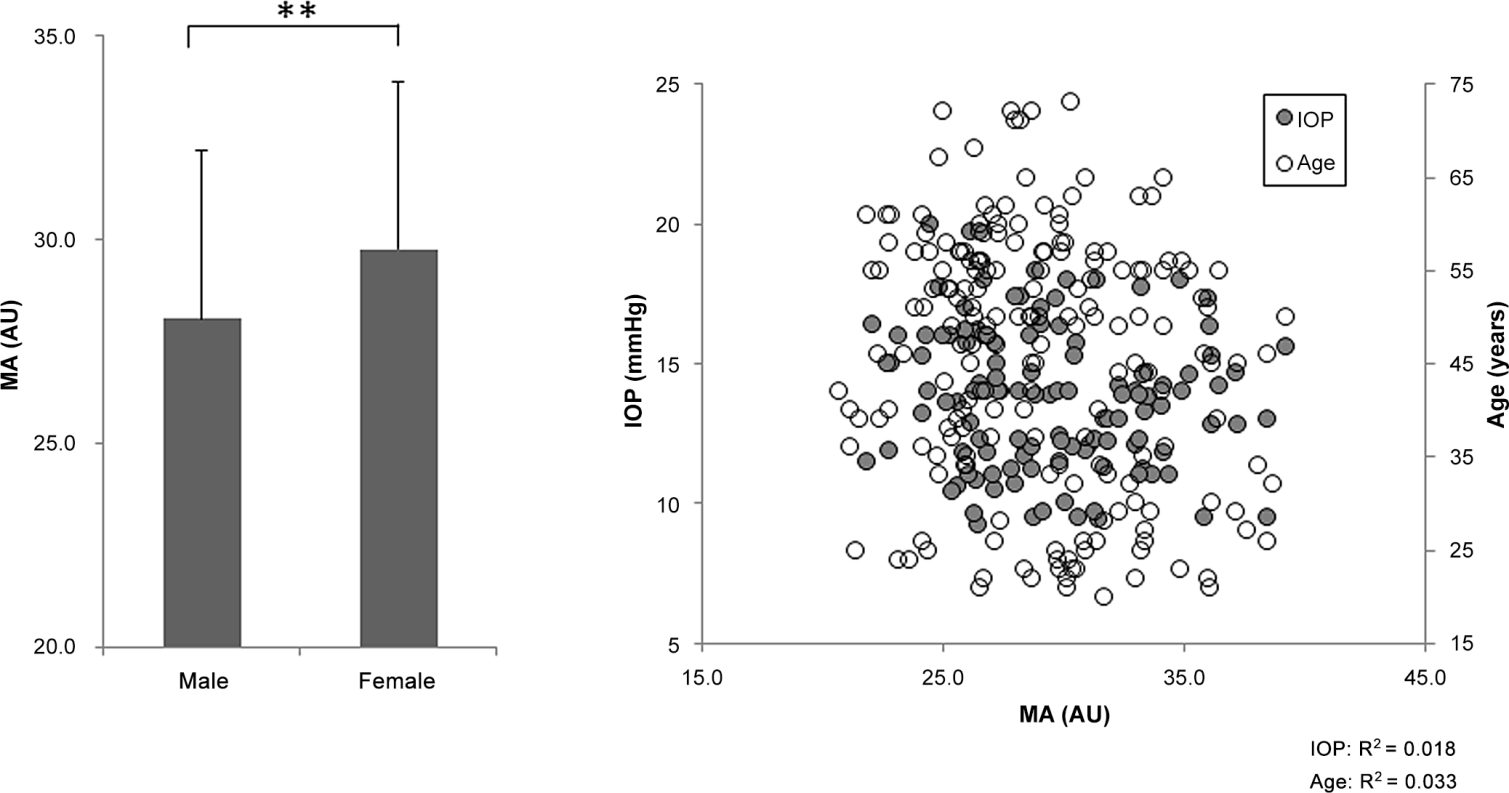
Relationship between overall mean blur rate (MA) and clinical findings. MA was higher in the female subjects than in the male subjects (*P* < 0.01, left). MA was not correlated with age or IOP (right).

One hundred and eighty-nine healthy subjects were divided into 5 age groups: 20–29, 30–39, 40–49, 50–59, and ≥ 60 YO; these groups included 36, 29, 34, 63 and 27 subjects, respectively. Each group had a statistically similar sex distribution (*P* = 0.233, Table 1). Overall, there was a trend towards decreased IOP and MV and increased SBP with age (*P* = 0.002, *P* = 0.035 and *P* = 0.006, respectively, Table 1). However, there were no age trends for SE, DBP, MT, or MA (*P* = 0.082, *P* = 0.283, *P* = 0.297, and *P* = 0.140, respectively, Table 1). MV was lower in the ≥ 60 YO group than in the 20-29-YO group (*P* = 0.007, Table 1), but there were no differences in MT or MA (*P* = 0.227 and *P* = 0.059, Table 1). IOP was also lower in the ≥ 60 YO group than in the 20-29-YO group (*P* = 0.007, Table 1). SBP was higher in the ≥ 60 YO group than the 20-29-YO group (*P* = 0.046, Table 1). Furthermore, there were age trends for IOP, MV and SBP (*P* = 0.002, *P* = 0.006 and *P* = 0.035, respectively, Table 1). MV was weakly correlated with age (r = -0.24, *P* < 0.001, Fig 1 right) and MT was negatively correlated with IOP (r = -0.29, *P* = 0.001, Fig 2 right). There were no correlations between MV, MT or MA and SE, SBP or DBP. MA and MV were higher in the female subjects than in the male subjects (*P* < 0.01, Fig 3 left and *P* < 0.05, Fig 1 left, respectively), while there was no such trend for MT (Fig 2 left). A series of multiple regression analyses of independent contributing factors revealed that sex, IOP and age contributed to MV (*P* < 0.001, *P* = 0.003 and *P* = 0.024, respectively, Table 2), IOP contributed to MT (*P* = 0.003, Table 3) and sex and IOP contributed to MA (*P* < 0.001 and *P* = 0.002, respectively, Table 4). SBP made no contribution to MA, MV, or MT (*P* = 0.979, *P* = 0.234, and *P* = 0.080, respectively, Tables 2–4).

**Table 2:** Multiple regression analysis for factors independently contributing to MV.

Variable			
Dependent	Independent	β	*P* value
MV	Age	-0.221	0.024
	Sex (F)	0.339	<0.001
	IOP	-0.286	0.003
	SE	-0.026	0.771
	SBP	0.156	0.234
	DBP	-0.051	0.691

β = standard partial regression coefficient, DBP = diastolic blood pressure, F = female, IOP = intraocular pressure, MV = mean blur rate in vessel area, SBP = systolic blood pressure, SE = spherical equivalent

**Table 3:** Multiple regression analysis for factors independently contributing to MT.

Variable			
Dependent	Independent	β	*P* value
MT	Age	0.150	0.136
	Sex (F)	0.147	0.124
	IOP	-0.294	0.003
	SE	0.066	0.466
	SBP	-0.238	0.080
	DBP	0.155	0.244

β = standard partial regression coefficient, DBP = diastolic blood pressure, F = female, IOP = intraocular pressure, MT = mean blur rate in tissue area, SBP = systolic blood pressure, SE = spherical equivalent

**Table 4:** Multiple regression analysis for factors independently contributing to MA.

Variable			
Dependent	Independent	β	*P* value
MA	Age	-0.189	0.050
	Sex (F)	0.377	<001
	IOP	-0.302	0.002
	SE	-0.038	0.664
	SBP	0.003	0.979
	DBP	0.094	0.463

β = standard partial regression coefficient, DBP = diastolic blood pressure, F = female, IOP = intraocular pressure, MA = overall mean blur rate, SBP = systolic blood pressure, SE = spherical equivalent

## Discussion

We analyzed variations in MBR in a population of normal individuals in order to determine the potential of MBR as a normative value for inter-individual and inter-group comparisons. We found that SBP increased with age but that IOP and MV decreased with age. Furthermore, MV differed significantly between the 20-29-YO subjects and the ≥ 60-YO subjects, although there were no differences in MA or MT. Additionally, while MA and MV tended to be higher in the female subjects than the male subjects, this was not true for MT. Finally, a series of multiple regression analyses of independent contributing factors revealed that sex and IOP contributed to MA, that sex, IOP and age contributed to MV, but that IOP was the only contributor to MT. SBP did not contribute to either MA, MV, or MT.

Our cross-sectional study supports previous research showing that IOP tends to decrease with age while SBP increases.[18,19] However, while previous reports have described a negative correlation between MA and age,[20,21] we found that this relationship was not significant in the current study (*P* = 0.05). Several other reports that used laser Doppler flowmetry to investigate blood flow and volume in the ONH of healthy subjects also found that it had a negative correlation with age.[22,23] Our study also supports earlier data showing that MT values are stable regardless of the age of the subject.[20] Interestingly, the multivariate analysis revealed a number of differences in factors to contributing to MV and MT, i.e., age, sex and IOP vs. IOP only, respectively. Though the exact reason is unclear, one possibility is related to measurement area: while the area measured by MV contains large vessels, the area measured by MT lacks these vessels and contains only capillaries. MT is thus less affected than MV by changes related to age-dependent atherosclerosis, and blood flow measured with MT is accordingly more stable with age.

Our observation that MA and MV were higher in female subjects than males was interesting, and is likely due to the lower rate of atherosclerosis in women than in men.[24,25] This would most directly affect MV, and then affect MA in turn, which was also confirmed recently.[14] Furthermore, previous reports have shown that cerebral blood flow is significantly higher in women than in men among normal adult individuals[26] and that the maintenance of cerebral blood flow speed during posture change and cerebral autoregulation is better in female subjects.[27] The existence of ocular blood flow autoregulation in response to posture change has also been confirmed in a study that used LSFG,[28] and although the mechanism underlying sex-based differences in ocular circulation and autoregulation remains unclear, we speculate that female hormones, such as estradiol, an estrogen, may affect ocular circulation, especially in the vessel area of the ONH.[29]

This study confirmed that MT was more stable with age and sex than MA or MV. This is an important new finding, as it shows that MT can serve as a normative value for inter-individual and inter-group comparisons. Currently, LSFG measurements are mainly used to monitor changes in ONH or choroid circulation over time at a single site in the same eye.[30] This limitation arises from the bias introduced to the laser speckle signal by fundus pigmentation.[31] Previous efforts to overcome this bias have relied on analyzing pulse waveform parameters of MBR, such as blowout time and falling rate. These waveform parameters were found to be comparable between individuals and to correlate with age.[20,32,33] Additionally, to answer the basic question of whether MBR itself is also an inter-individually comparable parameter, we recently completed a study in which we induced ONH ischemia in pigmented and albino rabbits and then compared measurements taken with MBR and CBF. Our report showed that MT was closely correlated with hydrogen gas clearance-measured CBF in albino and pigmented rabbits with or without chronic ischemia-induced ONH atrophy.[34] This implied that individual pigmentation has little effect on MT, and that MT should thus also be suitable for inter-individual comparisons. Furthermore, since the ONH is pigment-free in all human individuals, regardless of ethnicity, inter-individual comparisons of MT should be valid in all patients. Finally, we showed that relative flow volume, an index of blood flow derived from MBR, is an accurate and reliable index of blood flow speed and volume in the human retina.[35] Now, building on these previous reports, the findings reported here suggest that measurements of MT are stable independent of age or sex in the normal population, that MV decreases with the normal aging process and that MV differs between the sexes. The clear implication of all these recent findings, in particular that MT is stable, while MV is age- and sex-dependent, is that LSFG is a valuable tool to evaluate ocular blood flow and MBR values, and should enable valuable future comparisons of ONH circulation between individuals and groups, in addition to its current role in comparing circulation at a single site over time in an individual eye.

The earliest version of LSFG was introduced in the 1990s as a non-invasive method of imaging tissue circulation in the choroid and ONH.[16] Originally, LSFG imaging represented the difference between average speckle intensity in a series of scans and speckle intensity in each scan. This ratio, a quantitative index of blood speed, was defined as normalized blur (NB)[16], and was displayed as a colorized, two-dimensional map.[16] Later versions of LSFG replaced NB with square blur rate (SBR; the square of NB), and the current version uses MBR (SBR multiplied by two). Other improvements in modern LSFG systems include a much improved spatial resolution, due to a larger, 750 × 360-pixel, charge-coupled-device-based image sensor (in contrast to the 100 × 100-pixel, specialized sensor in NB-based LSFG)[17,36,37,38]and the use of eye-tracking.[39] Together, these changes have considerably broadened the usefulness of LSFG. Originally, LSFG was mainly used to monitor changes in blood circulation over time at a single site in the same eye, while interindividual comparisons relied on the analysis of pulse waveform parameters.[20,32] By contrast, the latest versions of LSFG, with their large measurement area and reduced measurement errors, should allow us to directly compare MBR, particularly MT, interindividually.[34]

Our study was somewhat limited by its nature as a cross-sectional retrospective case series enrolling only healthy Japanese subjects undergoing regular medical check-ups. Thus, we could not ensure that the subjects fasted or abstained from alcohol before the examination, and patients with a current smoking habit, which can affect blood circulation, might have been included in this study. However, the subjects were carefully selected in order to exclude those with a history of ocular disease, blood flow-affecting systemic disease requiring medical treatment, hypertension, hyperlipidemia, and diabetes. Therefore, since a number of existing papers, including a total of approximately 150 eyes, have provided normative values for age-dependent ocular changes measured with optical coherence tomography,[40,41] we consider that our results, which were obtained with a multiple regression analysis of 189 subjects, lend sufficient support to our conclusion: MV is closely related to age, sex and IOP, MT is stable independent of age or sex and is only related to IOP, and both are independent of SBP. Additionally, although MBR has previously been reported to decrease in glaucoma and ischemic ocular diseases,[7,9,42] this is the first report of a normative stable value for MT (13.5 ± 2.0 AU) over a wide age range. Nevertheless, although our findings promise to be useful in the clinical diagnostic and decision-making process, it may be advisable to use data gathered with our technique as supplementary information, similar to the "normative" IOP range of 10–21 mmHg.

In conclusion, our findings show that LSFG measurements of MBR are affected by IOP, regardless of which ONH region is measured, in a normal population. MV was affected by age and sex, and MT was stable independent of age or sex. Therefore, it should be possible to use LSFG measurements of MT to compare individuals or groups, and to make comparisons with nominal MT values. Thus, MT may be a quantitative, clinically useful way of identifying circulatory disturbances in ocular diseases. Further investigation is needed to determine the relationship between MBR and other clinical parameters, in order to establish MBR as a surrogate marker for the non-invasive, objective evaluation of ocular circulatory diseases or vascular aging.
